# Improving the Intrinsic Viscosity of Waste Polyester Fabrics via Controlled Micro-Glycolysis and Self-Polycondensation

**DOI:** 10.3390/polym18060727

**Published:** 2026-03-17

**Authors:** Rong Chen, Li-Bin Luo, Yu-Xin Lian, Xiao-Li Sun, Li-Ren Xiao

**Affiliations:** 1 College of Chemistry and Materials Science, Fujian Normal University, Fuzhou 350007, China; 2 Engineering Research Center of Polymer Green Recycling of Ministry of Education, Fujian Normal University, Fuzhou 350007, China; 3 College of Environmental and Resource Science, College of Carbon Neutral Modern Industry, Fujian Normal University, Fuzhou 350007, China

**Keywords:** polymer recycling, waste textile, polyester, controlled micro-glycolysis, self-polycondensation

## Abstract

Polyethylene terephthalate (PET) plays a pivotal role in the chemical fiber industry, constituting over 50% of fiber consumption. However, the reduction of the recycled fiber-derived viscosity of the PET significantly impacts its spinning performance and restricts its closed-loop recycling to high-value regenerated fibers. To address these limitations, this study explored the viscosity improvement of black and white waste fiber-derived polyester particles through a two-step process involving micro-glycolysis and self-polycondensation. Initially, a continuous micro-glycolysis of fiber-derived PET was carried out in a twin-screw extruder with ethylene glycol (EG), which effectively cleaves the ester bonds in the PET chains, generating oligomers with reactive hydroxyl end groups. Subsequently, these oligomers were repolymerized without purification, and a higher molecular weight regenerated PET with enhanced intrinsic viscosity was obtained with antimony ethylene glycolate (Sb-EG) as a catalyst. The results revealed that the intrinsic viscosity decreased exponentially with increasing EG dosage during glycolysis, reaching approximately 50% of the initial value at 0.2–2 phr EG dosages. Optimal viscosity enhancement was achieved at a polycondensation time of 1–3 h, resulting in improved thermal stability and reduced crystallization temperatures. Importantly, regenerated PET samples with EG dosages of ≤2 phr demonstrated intrinsic viscosities of about 0.70 dL/g, meeting the standard for spin-grade polyester fiber, which is used to produce regenerated polyester fibers. This recycling process is low cost, environmentally friendly, and easy to scale-up, contributing significantly to the development of industrial recycling of waste polyester fabrics.

## 1. Introduction

With the increasing production and consumption of textiles worldwide, the comprehensive utilization of solid waste from the textile and apparel industry has attracted growing attention [1]. Globally, the total amount of textile waste has reached approximately 92 million tonnes, with chemical fibers, such as polyester, nylon, and spandex, constituting the majority of this waste. Among them, polyester (polyethylene terephthalate—PET) fibers account for more than 50% [2]. Discarded PET textiles often contain dyes, pigments, spinning additives, and other contaminants, which significantly deteriorate the thermal stability, viscosity uniformity, and spinnability of the recycled materials [3]. Consequently, the development of efficient and high-value recycling technologies for waste polyester fibers has become a key challenge in advancing the sustainable management of textile solid waste [4,5,6].

Currently, the primary methods for recycling waste polyester fibers fall into two major categories: chemical recycling and mechanical recycling [7]. Chemical recycling includes glycolysis, hydrolysis, ammonolysis, pyrolysis, and so on [8]. Among them, glycolysis is the most widely employed method [9], particularly the alcoholysis reaction involving ethylene glycol. This process operates under mild conditions (180–240 °C, atmospheric pressure) and offers diverse catalyst options, including metal catalysts [10,11,12], metal oxide catalysts [13], ionic liquids [14,15,16], organic bases [17,18], nanoparticles [19,20], and others. Zhifeng Ao et al. synthesized surfactant-free ZnO nanoparticles (SF-ZnO) and applied them to the glycolysis of PET, and the PET conversion rate and BHET yield reached 100% and 97.3% respectively, demonstrating highly efficient catalytic performance and environmental friendliness [21]. Yachan Liu et al. developed a series of metal-free choline-based ionic liquids (ILs) as catalysts for the glycolysis of PET. Among them, choline acetate exhibited the best performance, offering catalytic activity and environmental benefits comparable to, or even surpassing, those of conventional imidazolium-based metal-containing ILs [15]. However, chemical recycling methods are typically batch solvent reactions, which suffer from disadvantages such as time-consuming, significant scale-up challenges, and high production costs. Additionally, a large amount of organic solvents used in the reaction and purification processes also increases the procedure of reuse and poses potential environmental pollution risks in scale-up production.

Mechanical recycling typically involves reprocessing waste polyester fibers through dehydration and pelletizing and is widely used due to its simplicity and cost-effectiveness. However, it still faces significant challenges regarding the degradation of mechanical properties and the low value. During mechanical recycling, challenges such as feeding difficulties and product heterogeneity often arise, resulting in regenerated materials with inferior mechanical performance [22]. Qu et al. investigated the thermomechanical degradation behavior of PET during repeated melt processing and reported that chain scission induced by thermal and shear stresses led to a significant reduction in molecular weight and intrinsic viscosity, thereby deteriorating the mechanical properties of the recycled PET [23]. Therefore, factors such as melting conditions, shear stress, and hydrolytic degradation during melt processing can substantially reduce the melt strength of recycled polyester, further deteriorating the product quality and limiting its range of applications.

Consequently, physicochemical recycling approaches have emerged as an effective alternative, among which the most widely adopted industrial process involves glycolytic depolymerization of PET using EG as the alcoholysis agent in a twin-screw extruder. However, current research has primarily focused on PET wastes derived from bottle flakes, industrial scraps, and food packaging materials [24]. In practice, approximately 60% of global PET production is used in the textile and apparel industry. Specifically, textile waste is typically in the form of loose, fibrous material, which complicates uniform feeding into reactors and extrusion systems, thereby reducing recycling efficiency [25]. Additionally, the high crystallinity further reduces the reactivity of fiber-derived PET wastes and slows down the alcoholysis reaction [26]. Gaozhe Liu et al. found that the depolymerization process of PET is slower and less efficient at higher crystallinities, due to the tightly packed, ordered molecular chains in crystalline regions, which hinder the accessibility of glycolysis agents like EG, requiring more energy for effective depolymerization [27]. Fiber-derived PET recycling is still a challenge.

In this study, we propose a two-step recycling strategy that integrates controlled micro-glycolysis of waste polyester fibers using EG as the alcoholysis agent in a twin-screw extruder and followed by self-polycondensation in a short time. This approach effectively enhances the intrinsic viscosity of the recycled fiber-derived PET, meeting the quality standards required for textile applications. Initially, discarded polyester fibers were pretreated and pelletized using a disc pelletizer to produce uniform recycled polyester pellets. This process effectively reduced the material’s crystallinity and transformed it from a fluffy state into a pelletized foam, facilitating subsequent processing. A comparative analysis was also conducted on two types of recycled PET: black PET (rPET-E) and white PET (rPET-w-E). rPET-E typically contains higher concentrations of dyes and other impurities, which can hinder the depolymerization reaction rate and affect the final properties. While rPET-w-E is less influenced by colorants, it still contains stabilizers, flame retardants, and other impurities that may interfere with the depolymerization process. Feeding these pellets into the twin-screw extruder for micro-glycolysis effectively overcomes the challenges of uneven feeding and material inhomogeneity, common in conventional recycling. The intense shear force generated by the twin-screw extruder ensures thorough mixing between EG and the polyester matrix, significantly shortening the reaction time, while the filter screen at the die head efficiently removes most solid impurities. In this process, ethylene glycol, a cost-effective and industrially mature alcoholysis agent, depolymerizes the polyester pellets into low-molecular-weight oligomers or monomers with a narrow molecular weight distribution and an increased number of reactive end groups, facilitating efficient chain-extension reactions during subsequent polycondensation. The glycolyzed intermediates were then transferred into a reactor, where Sb-EG was employed as the polycondensation catalyst to produce high-quality recycled PET granules with enhanced viscosity, fully meeting textile-grade specifications. This two-step extrusion–polycondensation strategy provides a promising pathway for the upcycling and high-value recycling of waste polyester fibers, enabling the production of textile-grade regenerated materials with improved performance.

## 2. Materials and Methods

### 2.1. Materials

Waste PET materials from the textile and apparel industry were sorted and shredded, followed by melt processing in a disc pelletizer to obtain black PET particles (rPET-F, which are from colored waste) and white PET particles (rPET-F-w, which are from white waste and do not contain dyes). These recycled PET particles were produced on a large scale by Fujian Baichuan Resource Recovery and Technology Co., Ltd, Fujian, China. Ethylene glycol (EG) was purchased from Energy Chemical (Shanghai, China), and antimony ethylene glycolate (Sb-EG) was obtained from Sinopharm Chemical Reagent Co., Ltd, Shanghai, China.

### 2.2. Micro-Glycolysis Process

In this experiment, the collected waste PET textiles (rPET-T) were pelletized using a disc pelletizer to obtain waste polyester particles, rPET-F and rPET-F-w. As shown in Table S1 and Figure S1, the crystallinity of rPET-T is 43.4% and the rPET-F is 27.5% after disc pelletizing. This is due to the effect of friction and temperature in disc pelletizing, making the fibers partially melt and bond together into lumps, which are then crushed into irregularly compacted particles. The obtained compacted particles also solved the feeding difficulties [3]. Both rPET-F and rPET-F-w were pre-dried in a vacuum drying oven at 120 °C for over 12 h to minimize moisture content. Subsequently, the pre-dried rPET-F or rPET-F-w were premixed with EG at the concentrations of 0, 0.2, 0.4, 0.6, 0.8, 1.0, 1.2, 1.4, 1.6, 1.8, 2, 4, and 6 phr, respectively. The mixtures were then introduced into a twin-screw extruder for micro-glycolysis. The temperature profile from screw zones 1 to 10 was set sequentially as 140, 165, 190, 230, 260, 268, 268, 265, 265, 260 °C, respectively. And the screw rotation speed was maintained at 300 rpm. The resultant glycolyzed polyester samples were designated as rPET-E (black) and rPET-w-E (white). The formulations of rPET-E and rPET-w-E samples are listed in Table 1.

### 2.3. Self-Polycondensation Process

rPET-E or rPET-w-E were uniformly blended with 200 ppm of Sb-EG catalyst and subsequently subjected to self-polycondensation in a high-temperature reactor. The reaction was conducted at a temperature of 268 ± 10 °C, under a vacuum of ≤200 Pa, with stirring at 750 rpm for 3 h. The resulting regenerated polyesters are designated as rPET-P (black) and rPET-w-P (white). The formulations are detailed in Table 2.

### 2.4. Characterizations

Thermogravimetric analysis (TGA) was performed using a Q50 thermogravimetric analyzer (TA Instruments, New Castle, DE, USA). Approximately 6–9 mg of each sample was weighed and heated from 35 °C to 700 °C at a constant heating rate of 10 °C/min under a nitrogen atmosphere.

Differential scanning calorimetry (DSC) was performed using a Q20 calorimeter (TA Instruments, New Castle, DE, USA). Approximately 6−8 mg of each sample was weighed and subjected to the following thermal cycle: initial heating from 30 °C to 280 °C at a rate of 10 °C/min, followed by a 5 min isothermal hold at 280 °C. The sample was then cooled to 30 °C at a rate of −10 °C/min and reheated to 280 °C at 10 °C/min.

Rheological measurements were carried out using a DHR-2 rotational rheometer (TA Instruments, New Castle, DE, USA). Rheological measurements were performed in stress-controlled mode using a rotational rheometer equipped with 25 mm diameter aluminum parallel-plate fixtures. The gap between the plates was set to 1 mm. Oscillatory frequency sweeps were conducted within the linear viscoelastic region of the material, under a fixed strain of 1.0%. All tests were carried out at 268 °C over a frequency range of 0.1 to 100 rad/s.

The intrinsic viscosity (*IV*) of the PET samples was determined using an Ubbelohde viscometer (Type 1835, Shanghai, China). Approximately 0.15 g of each sample was dissolved in 30 mL of a 1:1 (*w*/*w*) mixture of 1,1,2,2-tetrachloroethane and phenol. The solution was heated to 120 °C and stirred for 1.5 h to ensure complete dissolution. Flow times of the sample solution and the solvent were measured in a 25 °C oil bath. Each measurement was repeated three times, and the average value was used for calculation. The *IV* was calculated using the following Equation (1) [28]:

(1) $$IV=\frac{\sqrt{1+1.4(\frac{t_{1}}{t_{0}}\;-\;1)}-1}{0.35}$$

where $t_{1}$ is the flow time of the polymer solution, and $t_{0}$ is the flow time of the pure solvent.

The viscosity-average molecular weight ($M_{\eta }$) was calculated from the *IV* using the following Equation (2):

(2) $$M_{\eta }={\left(\frac{IV}{2.1\times 10^{-4}}\right)}^{\frac{50}{41}}$$

where $M_{\eta }$ is the viscosity-average molecular weight.

## 3. Results

**Figure 1:**
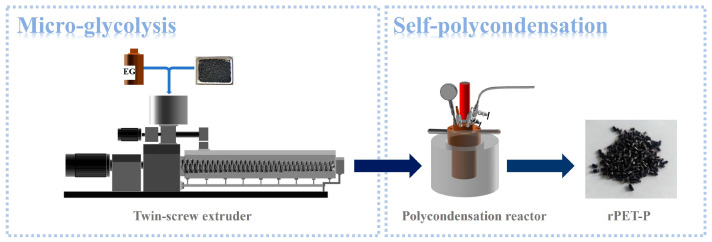
Schematic illustration of the micro-glycolysis and self-polycondensation process for preparing regenerated polyester from fiber-derived PET particle waste.

The recycling process of fiber-derived PET via micro-glycolysis and self-polycondensation involved two primary steps. Initially, rPET-F and rPET-F-w were blended with a specified amount of EG and underwent micro-glycolysis extrusion using a twin-screw extruder, yielding glycolyzed polyester samples rPET-E and rPET-E-w. Subsequently, these samples underwent self-polycondensation under high temperature and vacuum conditions in a polycondensation reactor to produce rPET-P and rPET-P-w with enhanced intrinsic viscosity. The detailed process is illustrated in Figure 1.

### 3.1. Micro-Glycolysis and Self-Polycondensation in Recycled Polyester Particles

**Figure 2:**
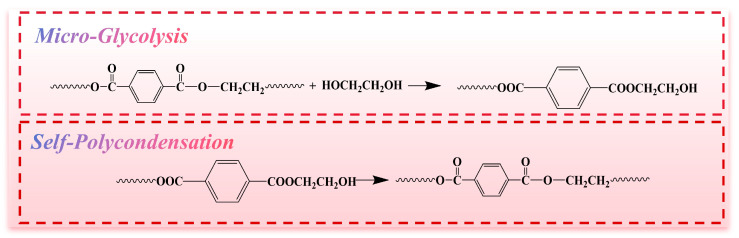
Schematic illustration of the micro-glycolysis and self-polycondensation reaction mechanism.

In the initial glycolysis stage, EG serves as a nucleophilic agent, selectively attacking and cleaving the ester linkages in the PET backbone. Due to the small amount of EG used, this reaction produces short-chain oligomers terminated with hydroxyl (–OH) end groups, thereby decreasing the intrinsic viscosity, enhancing melt flowability, and generating reactive chain ends for subsequent repolymerization. In the second stage, Sb-EG functions as a catalyst to accelerate intermolecular esterification. This promotes the formation of new ester bonds, driving step-growth polymerization and enabling molecular chain extension and viscosity recovery. However, excess EG that is not effectively removed may act as a chain terminator, limiting further polycondensation and reducing the overall efficiency of viscosity enhancement. The detailed reaction mechanism is illustrated in Figure 2.

### 3.2. Intrinsic Viscosity of Glycolyzed PET Particles

**Figure 3:**
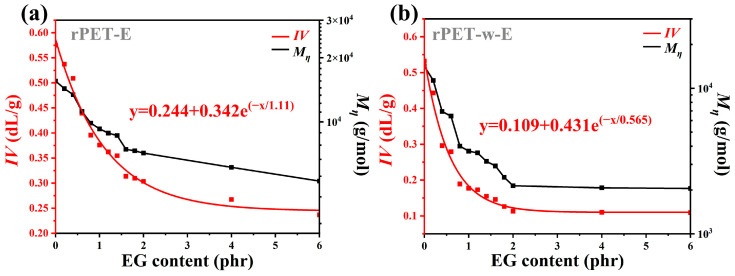
Exponential fitting curves of *IV* and $M_{\eta }$ for (**a**) rPET-E and (**b**) rPET-w-E.

Figure 3 presents the variation in *IV* and viscosity-average molecular weight ($M_{\eta }$) for rPET-E (a) and rPET-w-E (b) with EG dosage. As shown in Figure 3a, the intrinsic viscosity of rPET-E decreases markedly with increasing EG addition. Specifically, at EG content of 1 phr, the intrinsic viscosity decreases from 0.57 dL/g to 0.38 dL/g, representing 33.3% reduction relative to the untreated sample. When the EG dosage is increased to 2 phr, the intrinsic viscosity further decreases to 0.30 dL/g, corresponding to a 47.4% reduction. Beyond 2 phr, the decline in intrinsic viscosity becomes progressively less pronounced, indicating an approaching plateau. Concurrently, the intrinsic viscosity of rPET-E exhibits an exponential decrease with increasing EG concentration. At 2 phr EG, $M_{\eta }$ approaches approximately 7200 g/mol. The experimental data were fitted using a regression model, yielding a high correlation coefficient (R^2^ ≈ 1), suggesting a strong predictive relationship between EG content and the extent of molecular weight degradation. The fitted curve is shown below:

(3) $$y=0.244+0.342e^{\left(-\frac{x}{1.11}\right)}$$

where x represents the EG dosage (phr), and y denotes the *IV* (dL/g).

As illustrated in Figure 3b, the *IV* and $M_{\eta }$ of rPET-w-E samples exhibit a similar decreasing trend to that observed in rPET-E with increasing EG dosage. This trend is consistent with the findings of Wang et al., whose study also demonstrated that during the alcoholysis process, as the alcoholysis agent increased, the molecular weight and intrinsic viscosity of PET decreased rapidly. When the alcoholysis agent exceeded 10 wt%, the effect plateaued, indicating that the alcoholysis process had reached saturation [28]. Specifically, the *IV* of rPET-w-E decreases sharply from 0.53 dL/g to 0.18 dL/g at an EG dosage of 1 phr. When the EG dosage is further increased to 2 phr, the intrinsic viscosity drops to 0.11 dL/g. Beyond this level, the rate of decrease becomes progressively less pronounced, indicating a plateau-like behavior, with no significant viscosity reduction at higher EG dosages. A comparable trend is observed for the $M_{\eta }$. As the EG dosage increases, the $M_{\eta }$ of rPET-w-E decreases substantially, reaching approximately 2100 g/mol at 2 phr. The experimental data were fitted using a regression model, resulting in the following fitted curve:

(4) $$y=0.109+0.431e^{\left(-\frac{x}{0.565}\right)}$$

A comparison of the *IV* curves for glycolyzed black and white polyester particles reveals that, at the same EG dosage, rPET-w-E exhibits a lower intrinsic viscosity than rPET-E. Moreover, the reduction in *IV* for rPET-F-w is more pronounced, indicating that the white particles are more sensitive for the EG to chain scission under identical glycolysis conditions. It indicates that the presence of impurities in rPET-E influences the alcoholysis of PET. When the EG content is below 2 phr, both types of particles exhibit a substantial decrease in *IV* and a significant increase in melt fluidity. This confirms that the EG dosage is a critical parameter in regulating chain cleavage during the glycolysis process. Enhanced melt fluidity is advantageous for removing the embedded impurities from polyester particles. However, excessive fluidity can be detrimental to the following processing, particularly when the melt becomes too weak to be drawn and can only be conveyed. At EG dosages exceeding 2 phr, the depolymerization efficiency reaches a plateau, with no further substantial improvements. This saturation behavior suggests that most accessible ester linkages have already reacted. Additionally, excess EG may be lost due to evaporation under high processing temperatures or may not react effectively due to limited residence time in the twin-screw extruder, resulting in a nonlinear relationship between EG addition and its actual reactive availability in the melt.

Therefore, controlling the *IV* is essential to balance the glycolysis efficiency with downstream processability in the recycling of waste polyester materials. In this study, empirical models were established based on multiple experimental datasets, providing predictive equations for *IV*. These models can guide the selection of appropriate EG dosages based on specific product requirements, enabling precise and tunable glycolysis performance.

### 3.3. Thermal Properties of Glycolyzed Polyester Particles

**Figure 4:**
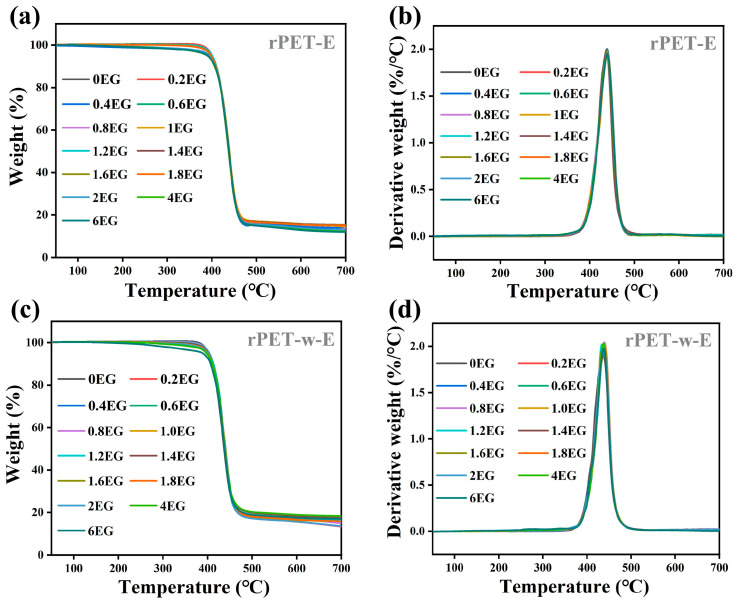
TGA curves of rPET-E and rPET-w-E (**a**,**c**); DTG curves of rPET-E and rPET-w-E (**b**,**d**).

Figure 4 presents the TGA and DTG curves of rPET-E and rPET-w-E samples glycolyzed with different dosages of EG, while Table 3 summarizes the corresponding thermal decomposition data. As shown, the T_d_ values of both rPET-E and rPET-w-E samples decrease slightly with increasing EG content. At an EG dosage of 2 phr, the T_d_ of rPET-E drops by 7.6 °C while that of rPET-w-E decreases by 3.8 °C, compared to their respective non-glycolyzed counterparts. Moreover, the DTG curves for each sample series are nearly superimposed. These results suggest that while glycolysis with increasing EG dosage leads to partial chain scission and a reduction in molecular weight, the overall decrease in thermal decomposition temperature remains minimal. This implies that the resulting glycolyzed products still retain sufficient molecular weight and exhibit good thermal stability.

**Figure 5:**
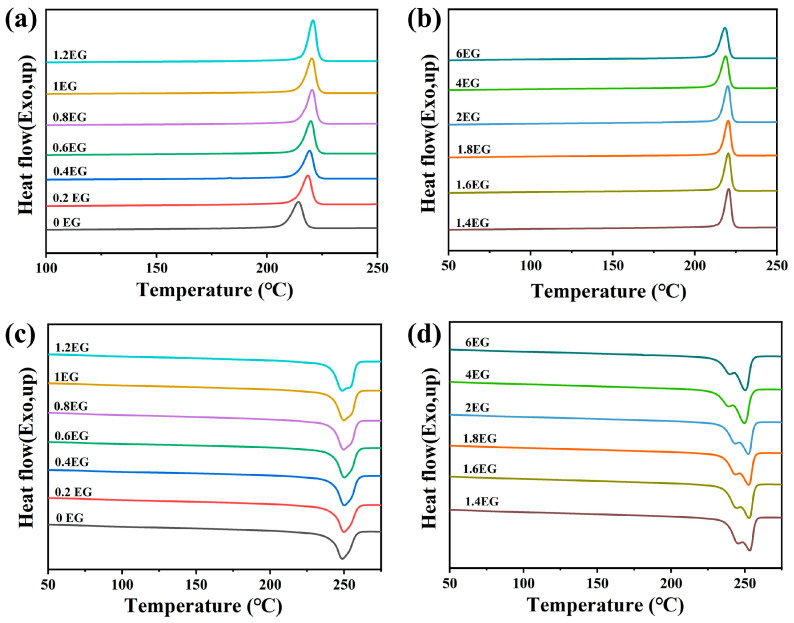
DSC curves of rPET-E samples prepared with varying EG dosages: second cooling curves (**a**,**b**) and third heating curves (**c**,**d**).

Figure 5 displays the DSC results for rPET-w-E samples glycolyzed with various dosages of EG, where Figure 5a,b show the second-cycle cooling curves and Figure 5c,d show the third-cycle heating curves. The corresponding thermal parameters, including crystallization temperature (T_c_), melting temperature (T_m_), and glass transition temperature (T_g_), are summarized in Table 4. In the cooling profiles, a noticeable increase in T_c_ is observed upon the addition of EG. Specifically, at 0.2 phr EG, T_c_ rises from 214 °C (pristine PET particles) to 218 °C. As the EG dosage increases further, T_c_ continues to climb, reaching 220 °C at 6 phr, representing an overall enhancement of 6 °C relative to the untreated sample. This behavior is attributed to the molecular changes induced by glycolysis, and the PET backbone undergoes partial depolymerization, generating short chains. These species not only enhance chain mobility but also serve as effective nucleation sites, thereby facilitating crystallization during cooling and elevating T_c_. In the subsequent heating scan, T_g_ decreases progressively with increasing EG content. Since T_g_ reflects the onset of chain segment mobility, this trend suggests reduced molecular weight and chain entanglement in the glycolyzed samples, requiring less thermal energy to transition from the glassy to the rubbery state. A similar trend was reported by Levente Kárpáti et al., it was observed that with an increase in the amount of adipic acid (AdAc), the T_g_ of the resulting unsaturated polyesters after glycolysis-polycondensation showed a significant decreasing trend [29]. Meanwhile, T_m_ exhibits a slight increase. The melting behavior evolves from a single peak in the non-glycolyzed sample to a distinct double-peak structure starting at 1.2 phr EG. With higher EG dosages, this dual-melting phenomenon becomes more pronounced. The lower temperature melting peak weakens, while the higher temperature peak intensifies.

This bimodal melting response is indicative of heterogeneous crystals. The lower T_m_ peak corresponds to the melting of imperfect or small crystallites, while the higher T_m_ peak is attributed to more stable and ordered crystals that may form during the heating process via melt-recrystallization. Such a dual-peak profile is characteristic of a melt–recrystallization–remelt mechanism [30]. As the EG dosage increases, the enhanced chain mobility promotes crystalline rearrangement, facilitating the formation of thermodynamically favorable crystalline domains.

**Figure 6:**
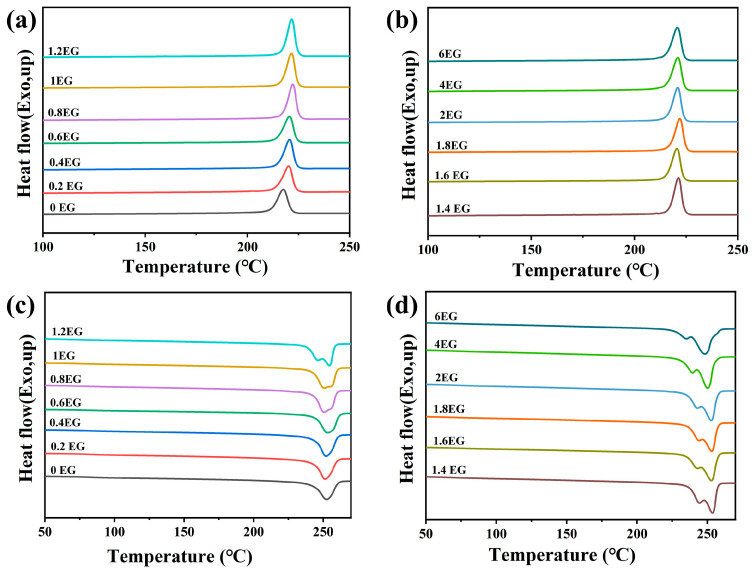
DSC curves of rPET-w-E samples prepared with varying EG dosages: second cooling curves (**a**,**b**) and third heating curves (**c**,**d**).

Figure 6 presents the DSC thermograms of rPET-w-E samples glycolyzed with different EG dosages. Figure 6a,b show the second-cycle cooling curves, while Figure 6c,d show the third-cycle heating curves. The corresponding thermal parameters, including T_m_, T_c_ and T_g_, are summarized in Table 5. As shown, T_c_ increases, reaching approximately 220 °C across all EG-treated samples. Notably, when the EG content exceeds 1 phr, the melting curves begin to exhibit shoulder peaks, indicating the presence of multiple crystals. As the EG dosage increases further, the temperature difference between the main melting peak and the shoulder peak becomes more pronounced. This behavior reflects the coexistence of less-ordered and more-ordered crystalline regions formed during the thermal history and recrystallization processes. Similar to the rPET-E samples, the introduction of EG leads to the cleavage of the ester bonds in the PET backbone, resulting in chain scission, molecular weight reduction, and enhanced segmental mobility.

### 3.4. Intrinsic Viscosity of Regenerated PET

**Figure 7:**
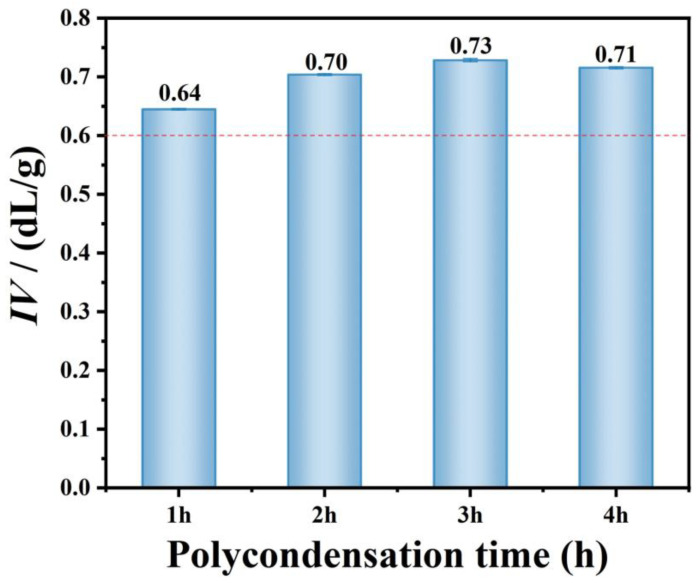
Intrinsic viscosity of regenerated polyester as a function of polycondensation time. The dashed line represents the minimum intrinsic viscosity required for polyester fiber spinning (0.60 dL/g).

To determine the optimal polycondensation time for intrinsic viscosity enhancement, a series of self-polycondensation trials were conducted using glycolyzed rPET-E1EG as the starting material. Figure 7 illustrates the *IV* of regenerated polyester as a function of polycondensation time. A clear trend is observed: the *IV* initially increases with reaction time, reaches a peak, and subsequently decreases. For textile applications, the minimum *IV* requirement for polyester fibers is typically >0.60 dL/g. When the polycondensation time varied from 1 to 4, all rPET samples exceeded this threshold, confirming their processability via melt-spinning. After 1 h of reaction, the *IV* reaches 0.64 dL/g and continues to increase, peaking at 0.72 dL/g at 3 h. However, extending the reaction time to 4 h leads to a noticeable decrease in intrinsic viscosity. This behavior is attributed to the competing mechanisms of chain growth and thermal degradation occurring simultaneously in the reactor. During the first 3 h, the polycondensation is dominant; beyond this point, prolonged exposure to high temperature promotes chain scission, leading to a net reduction in $M_{\eta }$ and *IV*. Therefore, in subsequent experiments, the reaction time was limited to a maximum of 3 h.

**Figure 8:**
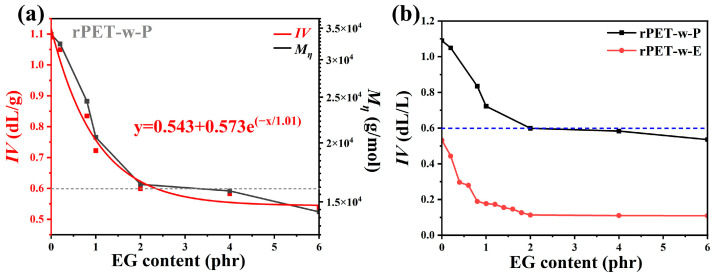
(**a**) Fitted curves of $M_{\eta }$ and *IV* for rPET-w-P samples glycolyzed with varying EG dosages and 3 h of self-polycondensation; (**b**) comparison of *IV* values for white PET particles samples before and after self-polycondensation with different EG dosages. The dashed line represents the minimum intrinsic viscosity required for polyester fiber spinning (0.60 dL/g).

Figure 8 shows the *IV* and $M_{\eta }$ for rPET-w-E samples glycolyzed with varying EG dosages after 3 h of self-polycondensation. As shown in Figure 8a, the *IV* of the regenerated PET decreases exponentially with increasing EG dosage after 3 h of self-polycondensation. This trend suggests that excessive chain scission caused by higher EG content limits the efficiency of molecular weight recovery. In Figure 8b, a significant increase in *IV* is observed after self-polycondensation of the glycolyzed samples. Specifically, the *IV* of the regenerated polyester derived from the 0.2 phr EG-treated rPET-w-E reaches 1.09 dL/g, while that of the sample glycolyzed with 1 phr EG increases to 0.72 dL/g. However, at an EG dosage of 6 phr, the *IV* of the regenerated polyester drops to 0.53 dL/g, indicating a diminished chain extension efficiency. This decline can be attributed to residual unreacted EG entrapped in the glycolyzed intermediate. During twin-screw extrusion, not all the EG is consumed when its dosage increases. The remaining EG acts as a chain terminator or sterically interferes with the polycondensation reactions, thereby limiting molecular weight growth and reducing the final *IV*. Therefore, strict control of EG dosage is essential in the micro-glycolysis and self-polycondensation process. An optimal balance must be achieved—sufficient EG should be used to enhance melt flow and facilitate impurity removal, but excessive EG must be avoided to prevent inhibition of polycondensation.

These results demonstrate that under well-regulated conditions, the “micro-glycolysis–self-polycondensation” strategy effectively enhances the *IV* of waste polyester. EG dosages in the range of 1–2 phr are optimal for achieving efficient chain scission and molecular weight recovery without compromising the quality of the regenerated polyester.

### 3.5. Thermal Properties of Regenerated Polyester Samples

**Figure 9:**
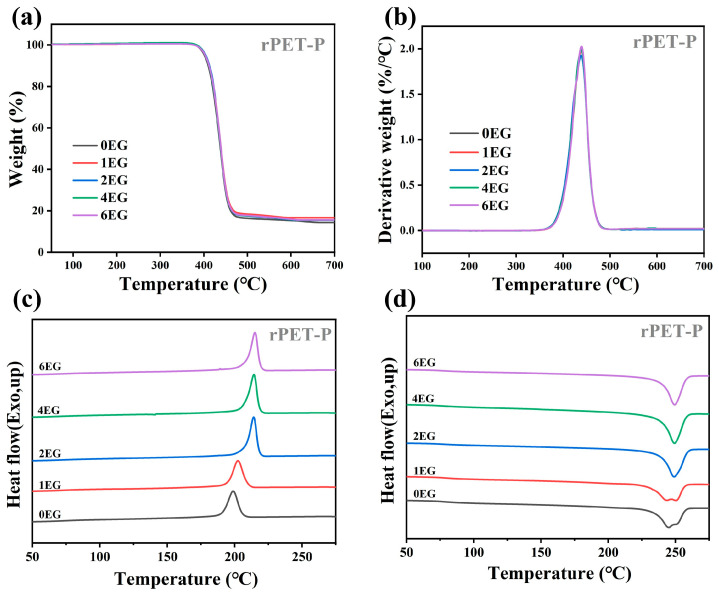
TGA curves (**a**,**b**) and DSC curves (**c**,**d**) of rPET-P samples obtained by self-polycondensation for 3 h with various EG loadings glycolysis.

Figure 9 displays the TGA curves (a), the DTG curves (b), the DSC cooling curves (c), and the heating curves (d) of rPET-P samples obtained by self-polycondensation for 3 h with various EG loadings glycolysis. The corresponding thermal parameters are summarized in Table 6. The TGA results reveal that the T_d_ of samples after polycondensation increase slightly compared to their glycolyzed counterparts. For example, at an EG dosage of 2 phr, the T_d_ increases from approximately 393.13 °C (glycolyzed sample) to 404.22 °C (after 3 h polycondensation), representing an enhancement of about 11 °C. This increase clearly indicates improved molecular weight and structural integrity during polycondensation, thereby enhancing the thermal stability of the regenerated PET. The DSC analyses show a notable decrease in T_c_ following polycondensation. Specifically, at 2 phr EG, T_c_ decreases by approximately 6 °C compared to the glycolyzed samples. This reduction may be attributed to increased molecular entanglement and chain length, which restricts the chain mobility and consequently retards the crystallization kinetics upon cooling.

Furthermore, T_m_ behaviors also significantly change after polycondensation. When EG dosages are in the range 2−6 phr, substantial chain scission during glycolysis is followed by efficient recombination and homogenization during polycondensation, resulting in a more uniform molecular weight distribution. Thus, the DSC heating curves exhibit a single melting peak, representing a homogeneous and stable crystalline structure. However, in the absence of glycolysis (EG content is 0 phr), the PET chains have difficulty undergoing polycondensation due to the lack of reactive end groups. The coexistence of short chains and long chains leads to different crystalline domains as shown in Figure 9d. When the EG content increases to 1 phr, the lower melting peak becomes smaller. Lastly, the T_g_ markedly increases after polycondensation, consistent with enhanced chain length and entanglement density, resulting in reduced segmental mobility and greater rigidity of the polymer matrix.

**Figure 10:**
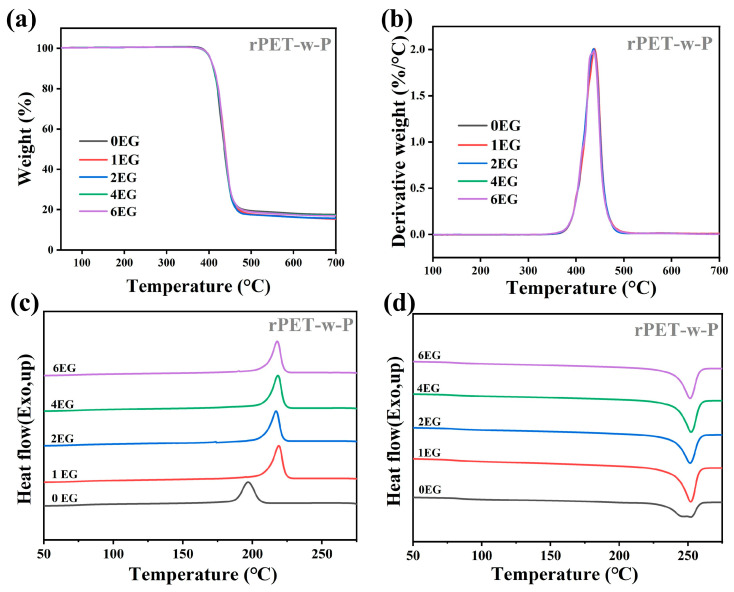
TGA curves (**a**,**b**) and DSC curves (**c**,**d**) of rPET-w-P samples obtained by self-polycondensation for 3 h with various EG loadings glycolysis.

Figure 10 presents the TGA curves (a), DTG curves (b), DSC cooling curves(c), and heating curves (d) of rPET-w-P samples obtained by self-polycondensation for 3 h with various EG loadings glycolysis. The corresponding thermal parameters are summarized in Table 7. The rPET-w-P samples exhibit trends analogous to those observed for the rPET-P counterparts. The TGA results indicate that self-polycondensation effectively facilitates chain extension and molecular restructuring, significantly enhancing the thermal stability of the material. For instance, at an EG dosage of 2 phr, the T_d_ increases from approximately 398.57 °C (glycolyzed) to 403.02 °C after polycondensation, representing an increment of about 4.5 °C. The DSC results similarly reveal a marked decrease in the T_c_ of the white regenerated PET after polycondensation. Specifically, at 2 phr EG dosage, the T_c_ drops by approximately 1.6 °C, implying enhanced molecular entanglement restricting chain mobility. Additionally, the T_m_ increases slightly, reflecting a more orderly and uniform crystalline structure, while the T_g_ exhibits a pronounced rise, indicative of increased molecular weight and restricted segmental mobility.

Overall, both black and white regenerated PET samples demonstrate improved thermal stability following polycondensation. However, the black PET samples exhibit a slightly greater enhancement in thermal stability than their white counterparts, which is likely due to differences in impurity content or the initial degradation level of the raw materials.

The regenerated PET pellets obtained after glycolysis and subsequent polycondensation were further processed via melt spinning at Fujian Baichuan Resource Recovery and Technology Co., Ltd. The rPET-P_1EG_ exhibited good spinning properties when applied to the production of regenerated polyester monofilaments and regenerated polyester fibers. The fiber-derived recycled PET can serve as feedstock for the production of regenerated polyester monofilaments. The tensile strength was 2.89 cN/dtex and the elongation at break was 38.3% for the monofilaments with a diameter of 0.74 mm tested according to the GB/T 14344-2008 standard [31]. Moreover, it can partially substitute recycled PET bottle flakes (rPET-B) at substitution ratios of 30% in the manufacture of recycled colored yarns without compromising the mechanical performance. The produced material was tested with the following result. The tensile strength and elongation at break 150 D of regenerated polyester fibers were 4.04 cN/dtex and 22.9%, respectively, according to the GB/T 14644-2008 standard. The detailed mechanical performance data are provided in the Supporting Information (SI). This result indicates the technical feasibility and industrial potential of converting waste polyester fibers into high-value textile materials, thereby providing a promising pathway for the high-value recycling and circular utilization of polyester textile waste.

### 3.6. Rheological Characterization of Regenerated Polyester Samples

**Figure 11:**
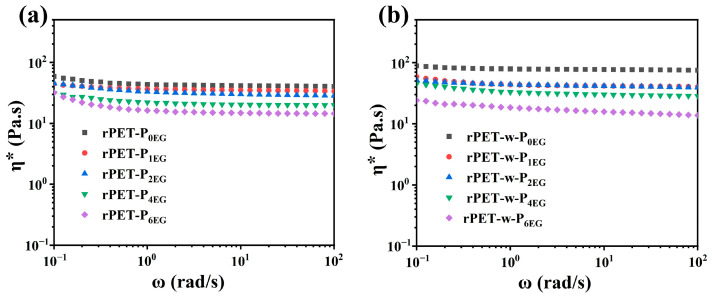
Complex viscosity as function of frequency for rPET-P_xEG_ (**a**) and rPET-w-P_xEG_ (**b**).

Figure 11 illustrates the relationship between complex viscosity (η*) and angular frequency (ω) for rPET-P (a) and rPET-w-P (b) samples prepared using varying EG amounts after polycondensation. The results indicate a clear reduction in complex viscosity with increased EG dosage. At lower EG dosages (0–2 phr), glycolysis results in limited chain scission, preserving the molecular weight and chain length of the PET samples. The intermediates retain long polymer chains and a high degree of end-group reactivity, which facilitate effective chain extension during the subsequent self-polycondensation process. This leads to the formation of a stronger molecular network, and consequently, the samples exhibit higher η*. This correlation demonstrates that maintaining longer polymer chains and high end-group reactivity enhances molecular entanglement and network formation, which is directly linked to the observed increase in viscosity. Therefore, the molecular architecture, characterized by longer chains and reactive ends, is a key determinant of the polymer’s rheological properties, influencing its flow behavior and processability. Conversely, higher EG dosages (4–6 phr) significantly increase the extent of polymer chain scission during glycolysis, leading to shorter oligomeric chains with lower molecular weights. These shorter chains have limited ability to extend during polycondensation. During the subsequent polycondensation, the residual unreacted EG further impedes efficient molecular recombination and chain growth, leading to a marked reduction in molecular weight. As the molecular weight decreases, its viscosity and processing stability diminish.

Overall, controlling the EG dosage (approximately 1 phr) proves essential for achieving optimal viscosity and processing performance in regenerated PET. This dosage strikes a balance between maintaining chain length and reactivity, which is crucial for achieving the desired molecular architecture and rheological properties.

## 4. Conclusions

This study successfully demonstrated an efficient approach to improving the viscosity of waste fiber-derived PET through micro-glycolysis and self-polycondensation. EG dosage is a key factor to balance macromolecular degradation and reconstruction, enhancing intrinsic viscosity, thermal stability, and processability. Specifically, a moderate EG dosage (~1 phr) was most effective in facilitating polymer reformation and achieving the desired viscosities, whereas excessive EG (≥2 phr) adversely impacted the chain growth and melt properties. Thereby, the relationship between PET IV and EG was constructed and used as guidance for the process parameter selection. Regenerated polyester monofilaments and regenerated polyester fibers were produced by melt-spinning from regenerated fiber-derived PET, and their mechanical properties met the required standards. These findings provide a viable route for upgrading recycled PET to meet textile industry standards, contributing significantly to sustainable waste management practices and resource recovery.

## Figures and Tables

**Table 1:** Formulation of glycolyzed polyester particle samples.

Sample	rPET-F (wt%)	rPET-w-F (wt%)	EG (phr)
rPET-E_1EG_	100	-	1
rPET-E_2EG_	100	-	2
rPET-E_4EG_	100	-	4
rPET-E_6EG_	100	-	6
rPET-w-E_1EG_	-	100	1
rPET-w-E_2EG_	-	100	2
rPET-w-E_4EG_	-	100	4
rPET-w-E_6EG_	-	100	6

**Table 2:** Formulation of regenerated polyester samples.

Sample	rPET-F (wt%)	rPET-F-w (wt%)	EG (phr)
rPET-P_1EG_	100	-	1
rPET-P_2EG_	100	-	2
rPET-P_4EG_	100	-	4
rPET-P_6EG_	100	-	6
rPET-w-P_1EG_	-	100	1
rPET-w-P_2EG_	-	100	2
rPET-w-P_4EG_	-	100	4
rPET-w-P_6EG_	-	100	6

**Table 3:** T_d_ and T_max_ of rPET-E and rPET-w-E samples.

Sample	rPET-E		rPET-w-E	
EG Content (phr)	T_d_ (°C)	T_max_ (°C)	T_d_ (°C)	T_max_ (°C)
0	400.75	438.43	402.37	437.24
0.2	400.60	439.34	402.35	438.42
0.4	400.78	439.54	402.56	439.08
0.6	400.16	439.06	403.79	442.32
0.8	399.23	439.63	402.96	438.61
1	400.63	440.01	401.36	433.34
1.2	400.69	439.34	401.65	433.58
1.4	398.64	438.45	400.44	437.37
1.6	398.47	439.17	400.03	439.73
1.8	397.92	438.79	399.55	440.34
2	393.16	438.62	398.57	438.82
4	390.61	438.56	397.52	437.3
6	389.68	438.50	398.19	437.84

**Table 4:** DSC data of rPET-E samples prepared with varying EG dosages.

Sample	T_m1_ (°C)	T_m2_ (°C)	T_c_ (°C)	T_g_ (°C)
0	248.45	-	214.23	75.57
0.2	249.83	-	218.51	76.08
0.4	250.00	-	219.27	75.77
0.6	250.26	-	219.81	76.35
0.8	249.78	-	220.47	76.40
1	249.96	-	220.32	75.77
1.2	252.91	248.80	220.84	72.17
1.4	253.28	245.65	220.58	69.69
1.6	252.85	244.15	220.36	66.81
1.8	252.49	243.60	220.31	67.07
2	252.34	243.36	220.08	69.04
4	249.67	239.04	220.72	63.20
6	250.57	239.20	220.36	61.29

**Table 5:** DSC data of rPET-w-E samples prepared with varying EG dosages.

Sample	T_m1_ (°C)	T_m2_ (°C)	T_c_ (°C)	T_g_ (°C)
0 EG	252.48	-	217.52	81.16
0.2 EG	251.43	-	220.16	80.90
0.4 EG	252.07	-	220.62	78.67
0.6 EG	253.44	-	220.53	78.78
0.8 EG	255.31	-	222.19	74.20
1 EG	255.18	-	221.50	77.61
1.2 EG	254.49	246.68	221.66	71.20
1.4 EG	253.65	244.55	221.25	72.56
1.6 EG	252.88	243.13	220.51	68.66
1.8 EG	253.13	244.08	221.91	73.81
2 EG	252.78	242.89	220.9	66.84
4 EG	250.27	239.34	220.94	63.54
6 EG	248.35	235.08	220.26	57.05

**Table 6:** TGA and DSC analysis of rPET-PxEG samples.

Sample	T_d_ (°C)	T_max_ (°C)	T_m1_ (°C)	T_m2_ (°C)
rPET-P_0EG_	401.23	438.17	244.73	250.89
rPET-P_1EG_	403.87	439.70	243.67	250.48
rPET-P_2EG_	404.22	439.06	-	249.05
rPET-P_4EG_	403.02	438.41	-	249.26
rPET-P_6EG_	403.23	439.07	-	249.38

**Table 7:** TGA and DSC analysis of rPET-w-PxEG samples.

Sample	T_d_ (°C)	T_max_ (°C)	T_m1_ (°C)	T_m2_ (°C)
rPET-w-P_0EG_	402.73	437.62	245.15	252.33
rPET-w-P_1EG_	402.90	438.17	-	251.97
rPET-w-P_2EG_	403.02	436.54	-	251.6
rPET-w-P_4EG_	402.80	436.98	-	252.33
rPET-w-P_6EG_	403.33	436.63	-	251.74

## Data Availability

The raw data supporting the conclusions of this article will be made available by the authors on request.

## Supplementary Materials

The following supporting information can be downloaded at: https://www.mdpi.com/article/10.3390/polym18060727/s1, Figure S1: DSC Curves of rPET-F and rPET-T. (a) Heating curve, (b) Cooling curve. Table S1: DSC Thermal Performance Parameters of rPET-F and rPET-T.

## References

[1] Shirvanimoghaddam K., Motamed B., Ramakrishna S., Naebe M. (2020). Death by Waste: Fashion and Textile Circular Economy Case. Sci. Total Environ..

[2] Chu J., Cai Y., Li C., Wang X., Liu Q., He M. (2021). Dynamic Flows of Polyethylene Terephthalate (PET) Plastic in China. Waste Manag..

[3] Luo L.B., Chen R., Lian Y.X., Wu W.J., Zhang J.H., Fu C.X., Sun X.L., Xiao L.R. (2024). Recycled PET/PA6 Fibers from Waste Textile with Improved Hydrophilicity by In-Situ Reaction-Induced Capacity Enhancement. Polymers.

[4] Allen R.D. (2019). Waste PET: A Renewable Resource. Joule.

[5] Ren T., Zhan H., Xu H., Chen L., Shen W., Xu Y., Zhao D., Shao Y., Wang Y. (2024). Recycling and High-Value Utilization of Polyethylene Terephthalate Wastes: A Review. Environ. Res..

[6] Cao F., Wang L., Zheng R., Guo L., Chen Y., Qian X. (2022). Research and Progress of Chemical Depolymerization of Waste PET and High-Value Application of Its Depolymerization Products. RSC Adv..

[7] Bharadwaj C., Purbey R., Bora D., Chetia P., Maheswari R.U., Duarah R., Dutta K., Sadiku E.R., Varaprasad K., Jayaramudu J. (2024). A review on sustainable PET recycling: Strategies and trends. Mater. Today Sustain..

[8] Conroy S., Zhang X.L. (2024). Theoretical insights into chemical recycling of polyethylene terephthalate (PET). Polym. Degrad. Stab..

[9] Enache A.C., Grecu I., Samoila P. (2024). Polyethylene Terephthalate (PET) Recycled by Catalytic Glycolysis: A Bridge toward Circular Economy Principles. Materials.

[10] Jeya G., Dhanalakshmi R., Anbarasu M., Vinitha V., Sivamurugan V. (2022). A Short Review on Latest Developments in Catalytic Depolymerization of Poly (ethylene Terephathalate) Wastes. J. Indian Chem. Soc..

[11] Xin J., Zhang Q., Huang J., Huang R., Jaffery Q.Z., Yan D., Zhou Q., Xu J., Lu X. (2021). Progress in the Catalytic Glycolysis of Polyethylene Terephthalate. J. Environ. Manag..

[12] Bohre A., Jadhao P.R., Tripathi K., Pant K.K., Likozar B., Saha B. (2023). Chemical Recycling Processes of Waste Polyethylene Terephthalate Using Solid Catalysts. ChemSusChem.

[13] Ghosal K., Advances C.N.J.M. (2022). Recent Advances in Chemical Recycling of Polyethylene Terephthalate Waste into Value Added Products for Sustainable Coating Solutions—Hope vs. Hype. Mater. Adv..

[14] Nosir M.A., Ortuño M.A. (2024). Catalytic Function of Ionic Liquids in Polyethylene Terephthalate Glycolysis by Molecular Dynamics Simulations. RSC Sustain..

[15] Liu Y., Yao X., Yao H., Zhou Q., Xin J., Lu X., Zhang S. (2020). Degradation of Poly(ethylene Terephthalate) Catalyzed by Metal-Free Choline-Based Ionic Liquids. Green Chem..

[16] Fang P., Zheng X., Zhang R., Xu J., Yan D., Zhou Q., Xin J., Shi C., Xia S., Lu X. (2023). Accurate Layer Spacing Matching of Polyoxometalate (POM) Anion-Based Ionic Liquids (ILs) to Promote PET Alcoholysis. ChemCatChem.

[17] Darai T.E., Ter-Halle A., Blanzat M., Despras G., Sartor V., Bordeau G., Lattes A., Franceschi S., Cassel S., Chouini-Lalanne N. (2024). Chemical Recycling of Polyester Textile Wastes: Shifting Towards Sustainability. Green Chem..

[18] Luo Y.-J., Sun J.-Y., Li Z. (2024). Rapid Chemical Recycling of Waste Polyester Plastics Catalyzed by Recyclable Catalyst. Green Chem. Eng..

[19] Vinitha V., Preeyanghaa M., Anbarasu M., Neppolian B., Sivamurugan V. (2023). Chemical Recycling of Polyester Textile Wastes Using Silver-Doped Zinc Oxide Nanoparticles: An Economical Solution for Circular Economy. Environ. Sci. Pollut. Res. Int..

[20] Yun L.X., Wu H., Shen Z.G., Fu J.W., Wang J.X. (2022). Ultrasmall CeO_2_ Nanoparticles with Rich Oxygen Defects as Novel Catalysts for Efficient Glycolysis of Polyethylene Terephthalate. ACS Sustain. Chem. Eng..

[21] Ao Z., Deng J., He W., Liu T., Wang J., Yang H., Shen Z., Chen J. (2024). Low-temperature One-Step Synthesis of Surfactant-Free ZnO Nanoparticles for Efficient Glycolysis of PET. Chem. Eng. J..

[22] Uzosike C.C., Yee L.H., Padilla R.V. (2023). Small-Scale Mechanical Recycling of Solid Thermoplastic Wastes: A Review of PET, PEs, and PP. Energies.

[23] Wu H., Lv S., He Y., Qu J.-P. (2019). The Study of the Thermomechanical Degradation and Mechanical Properties of PET Recycled by Industrial-Scale Elongational Processing. Polym. Test..

[24] Majumdar A., Shukla S., Singh A.A., Arora S. (2020). Circular Fashion: Properties of Fabrics Made from Mechanically Recycled Poly-Ethylene Terephthalate (PET) Bottles. Resour. Conserv. Recycl..

[25] Damayanti D., Wulandari L.A., Bagaskoro A., Rianjanu A., Wu H.-S. (2021). Possibility Routes for Textile Recycling Technology. Polymers.

[26] Enking J., Becker A., Schu G., Gausmann M., Cucurachi S., Tukker A., Gries T. (2025). Recycling Processes of Polyester-Containing Textile Waste–a Review. Resour. Conserv. Recycl..

[27] Liu G., Zuo W., Hao M., Zhu K., Wang F., Chen L. (2025). The Influence of Crystallinity on the Depolymerization Mechanism of PET Fibers. Polym. Degrad. Stab..

[28] Wang H., Wei X., Zheng W., Tang H., Xi Z., Sun W., Zhao L. (2025). Controlled Depolymerization of Polyethylene Terephthalate Based on Twin-Screw Extruder and Repolymerization of the Depolymerized Products. Chem. Eng. Sci..

[29] Kárpáti L., Fogarassy F., Kovácsik D., Vargha V. (2019). One-Pot Depolymerization and Polycondensation of PET Based Random Oligo- and Polyesters. J. Polym. Environ..

[30] Ronkay F., Slezak E., Gere D., Lukacs N., Gyalai-Korpos M., Molnar A.D., Bocz K. (2025). Thermoanalytical Approach to Assess Riverine PET Litter and Its Recycling Potential. Sci. Rep..

[31] (2008). Testing Method for Tensile of Man-Made Filament Yarns.

