# Supplementary material for: Improving the Intrinsic Viscosity of Waste Polyester Fabrics via Controlled Micro-Glycolysis and Self-Polycondensation
Source: Polymers (Basel). 2026 Mar 17;18(6):727. doi: 10.3390/polym18060727 (PMC13030470; doi:10.3390/polym18060727)
Supplement: Supplementary file 1 [file polymers-18-00727-s001.zip › polymers-4192140-supplementary.pdf]

Supporting Information

Improving the Intrinsic Viscosity of Waste Polyester Fabrics via Controlled Micro-Glycolysis and Self-Polycondensation

Rong Chen[1,3], Li-Bin Luo[1,3], Yu-Xin Lian[2,3], Xiao-Li Sun[2,3,*], Li-Ren Xiao[1,3,*]

[1] College of Chemistry and Materials Science, Fujian Normal University, Fuzhou 350007, China
[2] College of Environmental and Resource Science, College of Carbon Neutral Modern Industry, Fujian Normal University, Fuzhou 350007, China
[3] Engineering Research Center of Polymer Green Recycling of Ministry of Education, Fujian Normal University, Fuzhou 350007, China;
* Correspondence: sunxiaoli@fjnu.edu.cn(X.-L. S.); xlr1966@fjnu.edu.cn(L.-R. X.)

Academic Editor: Firstname Lastname

Received: date
Revised: date
Accepted: date
Published: date

Copyright: © 2026 by the authors. Submitted for possible open access publication under the terms and conditions of the Creative Commons Attribution (CC BY) license.

1. Crystallinity of Waste PET Fibers and Regenerated PET Pellets

The collected Waste PET textiles (rPET-T) were pelletized using a disc pelletizer to obtain regenerated polyester particles (rPET-F). The crystallinity of the samples was measured by Differential Scanning Calorimetry (DSC) using a Q20 calorimeter (TA Instruments, New Castle, DE, USA). Approximately 6–8 mg of each sample was weighed and subjected to the following thermal cycle: initial heating from 30°C to 280°C at a rate of 10°C/min, followed by a 5-minute isothermal hold at 280°C. The sample was then cooled to 30°C at a rate of -10°C/min and subsequently reheated to 280°C at 10°C/min. The results showed that the crystallinity decreased from 43.40% to 27.65%, indicating that the pelletization process effectively reduced the crystallinity, facilitating subsequent processing. Specific data are listed in Table S1, and the DSC heating and cooling curves are shown in Figure S1.

Table S1. DSC Thermal Performance Parameters of rPET-F and rPET-T

Sample	T_g (°C)	T_c (°C)	T_m (°C)	X_c(%)
rPET-T	81.70	197.92	252.96	43.40
rPET-F	80.84	206.66	250.48	27.65

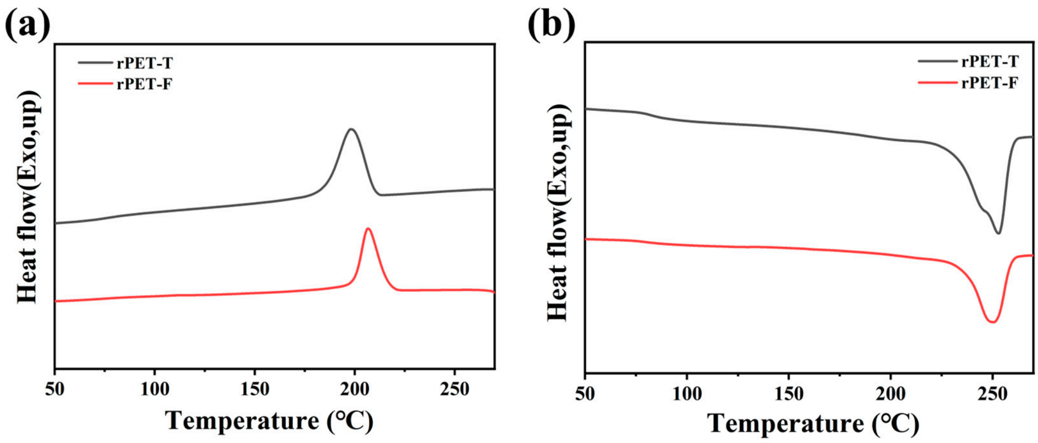

Figure S1. DSC Curves of rPET-F and rPET-T.(a) Heating curve, (b) Cooling curve

2. Mechanical Properties of Recycled Polyester Fiber

The polyester particles (rPET-F) was mixed with 1 phr ethylene glycol (EG) and fed into a twin-screw extruder for micro-glycolysis. Subsequently, the obtained product was subjected to polycondensation at 270 °C for 3 h to produce regenerated polyester pellets (rPET-P). The rPET-P was then sent to Fujian Baichuan Resource Recovery and Technology Co., Ltd. for melt spinning to produce regenerated polyester monofilaments and regenerated polyester fibers.

100% rPET-P was used as raw materials to produce regenerated polyester monofilaments. Fisrtly, rPET-P melt was transported to the metering pump through pipelines and extruded through the spinneret to form stable filaments. The filaments were then cooled and solidified in a water bath and subjected to cold drawing using the first set of drawing rollers. Subsequently, the monofilaments underwent hot-air drawing through the second oven and drawing rollers, followed by heat setting

through the third oven and drawing rollers before being wound to obtain regenerated polyester monofilaments. Mechanical properties of the monofilaments with a diameter of 0.74 mm were tested according to the GB/T 14344-2008 standard. The tensile strength was 2.89 cN/dtex and the elongation at break was 38.3%, with coefficients of variation of 1.56% and 7.99% for tensile strength and elongation at break, respectively.

For the production of regenerated polyester fibers, rPET-P was used to partially replace recycled PET bottle flakes (rPET-B). When rPET-P replaced 30% of rPET-B, melt spinning was performed under the following conditions: the spinning box temperature was controlled at 285–290 °C, the component pressure was maintained below 18 MPa, the quenching air temperature was controlled at 20–24 °C, and the POY winding speed was 3080 m/min. The produced 150D regenerated polyester fibers was tested according to the GB/T 14344-2008 standard. The tensile strength and elongation at break were 4.04 cN/dtex and 22.9%, respectively, with coefficients of variation of 2.44% and 5.01%.

Overall, the mechanical properties of both the regenerated polyester monofilaments and regenerated polyester fibers meet the standard requirements. The regenerated polyester monofilaments were used to produced the zippers and the regenerated polyester fibers were used to produce renewable fabrics in Fujian Baichuan Resource Recovery and Technology Co., Ltd.. These results demonstrate that recycled polyester derived from waste fibers can be effectively used for the production textiles, which provides a promising pathway for the high-value recycling and circular utilization of polyester textile waste.
